# The Pannexin 1 Channel and the P2X7 Receptor Are in Complex Interplay to Regulate the Release of Soluble Ectonucleotidases in the Murine Bladder Lamina Propria

**DOI:** 10.3390/ijms24129964

**Published:** 2023-06-09

**Authors:** Mafalda S. L. Aresta Branco, Alejandro Gutierrez Cruz, Lauren E. Peri, Violeta N. Mutafova-Yambolieva

**Affiliations:** Department of Physiology and Cell Biology, School of Medicine, University of Nevada Reno, Reno, NV 89557, USA; mafaldad@unr.edu (M.S.L.A.B.); agutierrezcruz@unr.edu (A.G.C.); lokane@med.unr.edu (L.E.P.)

**Keywords:** bladder, lamina propria, purinergic signaling, extracellular ATP, P2X7 receptor, pannexin 1, ectonucleotidases

## Abstract

The bladder urothelium releases ATP into the lamina propria (LP) during filling, which can activate P2X receptors on afferent neurons and trigger the micturition reflex. Effective ATP concentrations are largely dependent on metabolism by membrane-bound and soluble ectonucleotidases (s-ENTDs), and the latter are released in the LP in a mechanosensitive manner. Pannexin 1 (PANX1) channel and P2X7 receptor (P2X7R) participate in urothelial ATP release and are physically and functionally coupled, hence we investigated whether they modulate s-ENTDs release. Using ultrasensitive HPLC-FLD, we evaluated the degradation of 1,*N*^6^-etheno-ATP (eATP, substrate) to eADP, eAMP, and e-adenosine (e-ADO) in extraluminal solutions that were in contact with the LP of mouse detrusor-free bladders during filling prior to substrate addition, as an indirect measure of s-ENDTS release. Deletion of *Panx1* increased the distention-induced, but not the spontaneous, release of s-ENTDs, whereas activation of P2X7R by BzATP or high concentration of ATP in WT bladders increased both. In *Panx1^−/−^* bladders or WT bladders treated with the PANX1 inhibitory peptide ^10^Panx, however, BzATP had no effect on s-ENTDS release, suggesting that P2X7R activity depends on PANX1 channel opening. We concluded, therefore, that P2X7R and PANX1 are in complex interaction to regulate s-ENTDs release and maintain suitable ATP concentrations in the LP. Thus, while stretch-activated PANX1 hinders s-ENTDS release possibly to preserve effective ATP concentration at the end of bladder filling, P2X7R activation, presumably in cystitis, would facilitate s-ENTDs-mediated ATP degradation to counteract excessive bladder excitability.

## 1. Introduction

Urothelial ATP is released into the suburothelium/lamina propria (LP) during bladder filling [1,2], and it is thought to activate P2X receptors on afferent neurons residing in the urothelium or LP in order to convey the sensation of bladder fullness and initiate the micturition reflex [3,4]. Effective extracellular concentrations of ATP at its receptors are determined by both release and metabolism of ATP. In the LP of the bladder, sequential hydrolysis of ATP to ADP, AMP, and adenosine (ADO) is a mechanosensitive process mediated by both membrane-bound and soluble ectonucleotidases (s-ENTDs) [5]. While membrane-bound nucleotidases are expressed ubiquitously in most cell types, s-ENTDs conceivably have more specialized locations and functions [6,7], making them attractive as potential therapeutic targets. However, little is known about the regulatory mechanisms involved in the release of s-ENTDs.

Pannexin 1 (PANX1) channels and P2X7 receptors (P2X7R) participate in urothelial ATP release and signaling [8,9] and have been shown to interact both physically and functionally in several cell types and tissues [8,10,11,12,13,14,15,16]. This raises the question of whether PANX1 and P2X7R in the bladder mucosa also contribute to regulating the release of s-ENTDs.

PANX1 shares limited sequence homology with invertebrate innexin gap junction proteins. Unlike connexins and innexins, PANX1 proteins do not form gap junctions in normal physiological conditions, but instead, form single channels at the surface of the plasma membrane [17]. PANX1 channels can be activated by several stimuli, including mechanical stress [9,18], changes in voltage [10,19,20], via ligand binding receptors [21], increase in intracellular calcium [22,23], redox potential changes [24], oxygen deprivation [25,26], increase in extracellular potassium [11,26,27], tyrosine phosphorylation by Src kinase [19,28,29], and cleavage of the C terminal amino acids by caspases [30,31]. Although it is generally accepted that ATP release can be mediated through the PANX1 channel, it has been reasoned that channel permeability to ATP only occurs in response to particular stimuli, such as low oxygen, mechanical stress, or elevated extracellular potassium, whereas in response to voltage activation, PANX1 becomes highly selective for chloride and has very little ATP permeability [32]. Although the conformational state permeable to ATP remains to be resolved, it has been proposed that PANX1 may assume different conformational and conductance states, depending on the mechanism of activation or experimental conditions [33]. PANX1 contributes to a variety of physiological and pathological processes, such as the function of sensory neurons, modulation of synaptic processes, glucose uptake in insulin-stimulated adipocytes, regulation of blood flow, skeletal muscle contraction, acute and chronic liver diseases, inflammation, and cell death [34,35,36].

P2X7R is an ionotropic receptor that belongs to the P2X purinoceptor family distributed across several tissue types, including the bladder [37]. Activation of P2X7R requires 10–100-fold higher concentrations of ATP than other purinoreceptors (P2X7R EC_50_ by ATP ~100 µM), and results in the influx of sodium and calcium and outflow of potassium [38]. In the presence of physiological concentrations of calcium and magnesium, activation of P2X7R may require concentrations of ATP in the lower mM range [39,40]. As the cytosolic concentrations of ATP are 1–5 mM [41,42], it is conceivable that ATP released through exocytosis or through PANX1 channels in close proximity with P2X7R can transiently reach effective concentrations to activate this receptor. Like PANX1, P2X7R plays a role in activating and sustaining inflammation and has been implicated in several inflammatory and autoimmune diseases and cancer [43,44], including in the inflammatory processes of animal models of cyclophosphamide or acrolein-induced cystitis [45,46]. It was initially proposed that activation of P2X7R permeabilizes cells to large molecules, such as nucleotides, through gradual dilation of its own channel pore [38,47], or via interaction with other channel-forming proteins (e.g., PANX1) [10]. However, the pore dilation hypothesis has been challenged by recent data, which suggest that these findings likely reflect errors in the methodologies used [48,49]. Rather, it has been argued that permeability to sodium, calcium, potassium, and higher-molecular-mass organic molecules (NMDG^+^ or spermidine^3+^) occurs simultaneously and that additional permeation routes through interaction with other auxiliary pore-forming proteins, such as PANX1, may exist [48,49].

It has been proposed that P2X7R activation and subsequent increase in intracellular calcium [22] or phosphorylation by Src kinases [19] can activate the PANX1 channel, thus stimulating further release of ATP and sustaining P2X7R activation. This feed-forward mechanism of ATP-induced ATP release may lead to cell death if left unchecked. Interestingly, some safeguard mechanisms seem to regulate the PANX1-P2X7R crosstalk and thus prevent overstimulation-induced cell death. First, high concentrations of ATP, but not ADP, AMP, or adenosine, have been shown to inhibit PANX1 currents [50,51]. Second, ATP promotes PANX1-P2X7R clustering and internalization in a concentration- and time-dependent manner. Moreover, modulation of endogenously released ATP through the addition of apyrase or inhibition of endogenous nucleotidases abolishes or increases PANX1-P2X7R clustering and internalization, respectively [14,52]. Finally, PANX1 attenuates P2X7R receptor-mediated calcium influx in various cell types through its C-terminal tail, and this effect is independent of the ion flux through PANX1 [53].

Thus, considering their important role in purinergic signaling, we investigated whether PANX1 and P2X7R may, either individually or through cross-talk, modulate the spontaneous and distention-evoked release of s-ENTDs in the LP of the bladder and, consequently, the degradation of ATP at rest or during bladder filling.

## 2. Results

### 2.1. Effect of PANX1 on the Release of s-ENTDs in the Bladder LP

#### 2.1.1. Panx1 Deletion Increases the Distension-Induced Release of s-ENTDs in the LP

**Figure 1:**
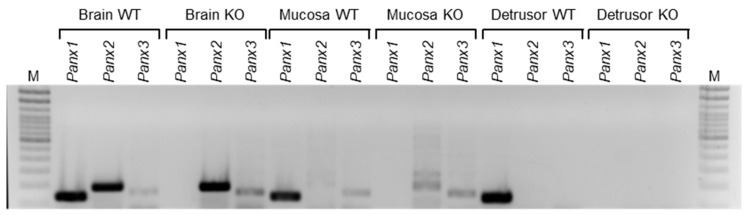
End-point PCR analysis of brain, bladder mucosa, and detrusor homogenates from wild-type (WT) (*n* = 1) and *Panx1*^−/−^ (*n* = 1) mice. M, molecular marker (100 bp ladder).

**Figure 2:**
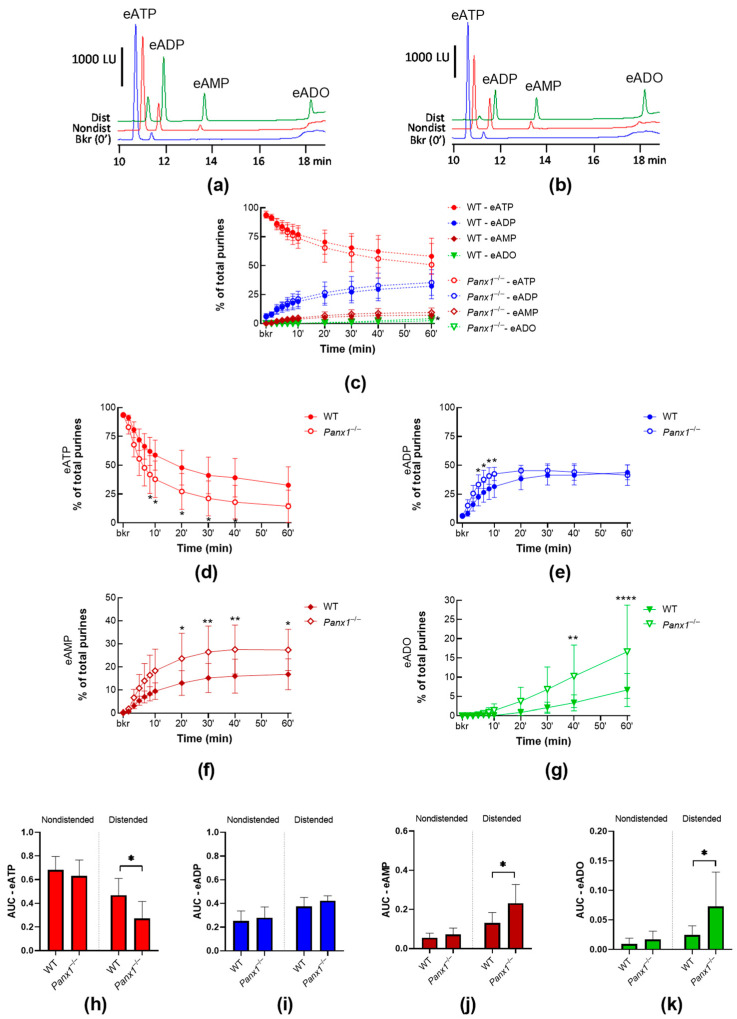
Degradation of eATP by s-ENTDs released in cELS of bladder preparations from WT and *Panx1^−/−^* mice. Original chromatograms of eATP in a beaker (Bkr, blue, 0′, no enzyme present) and at 60 min of enzymatic reaction in cELS of nondistended (Nondist, red) and distended (Dist, green) bladder preparations from WT (**a**) or *Panx1^−/−^* (**b**) mice. LU, luminescence units. Summarized data showing time courses of the degradation of eATP in cELS of nondistended (**c**) and distended (**d**–**g**) WT (*n* = 8–9) and *Panx1^−/−^* (*n* = 7) bladder preparations; *n*, number of bladder preparations. eATP substrate, and eADP, eAMP, and eADO products are represented as percentages of total purines (eATP + eADP + eAMP + eADO) present in reaction solutions at each time point. Asterisks denote significant differences from WT. * *p* < 0.05, ** *p* < 0.01,**** *p* < 0.0001; two-way ANOVA with Sidak’s multiple comparisons test. Mean area under the curve (AUC) values for time courses of eATP (**h**), ADP (**i**), AMP (**j**), and ADO (**k**). Asterisks denote significant differences from WT. * *p* < 0.05; unpaired *t* test.

To determine whether the PANX1 channel plays a role in regulating the release of s-ENTDs, we assessed the degradation of eATP in concentrated bathing solutions (cELS) of detrusor-free bladder preparations isolated from *Panx1^−/−^* mice and compared it with the degradation of eATP in cELS of WT mice. The complete absence of *Panx1* in the bladder mucosa was validated by end-point PCR. As shown in Figure 1, *Panx1* transcripts were detected in the brain, bladder mucosa, and detrusor homogenates from WT mice. In contrast, the *Panx1^−/−^* mouse line lacked the *Panx1* transcript entirely. *Panx1* deletion had no effect on the degradation of eATP in cELS of nondistended bladders when compared with WT (Figure 2c,h), whereas it significantly increased the degradation of eATP in cELS of distended bladders (AUC_WT_ 0.47 ± 0.14 vs. AUC_Panx1−/−_ 0.27 ± 0.14; *p* < 0.05) (Figure 2d,h). Thus, the substrate (i.e., eATP) decrease and product (i.e., eADP, eAMP, and eADO) increase (Figure 2d–k) were greater in cELS of *Panx1^−/−^* bladder preparations than in WT bladders. Note that from 8 min until the end of reactions, roughly 20% more eATP was hydrolyzed in cELS of *Panx1^−/−^* than in WT preparations. The increase in eADP in cELS of *Panx1^−/−^* bladders was transiently higher than in cELS of WT preparations, and the increase in eAMP and eADO was significantly higher (*p* < 0.05) in cELS of *Panx1^−/−^* than WT bladders in the last 40 and 20 min of reaction, respectively. These data indicated that deletion of *Panx1* results in increased release of s-ENTDs causing accelerated hydrolysis of eATP. Therefore, distention-induced activation of PANX1 should inhibit the release of s-ENTDs into the LP.

#### 2.1.2. ^10^Panx Has no Effect on the Release of s-ENTDs in the LP

**Figure 3:**
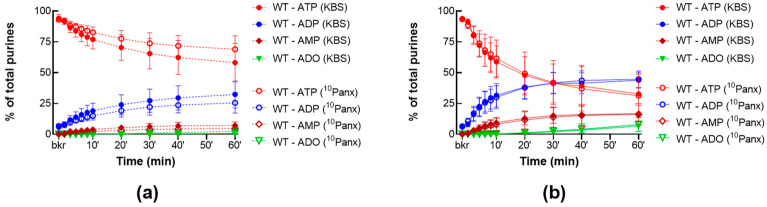
Effect of ^10^Panx on the eATP metabolism by s-ENTDs released in cELS. Summarized data showing time courses of the degradation of eATP in cELS in the presence of the vehicle (KBS, *n* = 8–9) or ^10^Panx (200 μM, *n* = 7) in nondistended (**a**) and distended (**b**) WT bladders; *n*, number of bladder preparations. eATP, eADP, eAMP, and eADO are shown as percentages of total purines (eATP + eADP + eAMP + eADO) present in reaction solutions at each time point for the duration of 1 h.

We next examined whether pharmacological inhibition of PANX1 would have the same effect as deletion of the *Panx1* gene. As shown in Figure 3, ^10^Panx1 at a concentration of 200 µM, failed to substantially alter the degradation of eATP in cELS of nondistended (Figure 3a) and distended (Figure 3b) preparations, suggesting that the release of s-ENTDs remained unchanged in the presence of ^10^Panx. 

### 2.2. Role of P2X7R in the Release of s-ENTDs in LP

#### 2.2.1. P2X7R Inhibition Does Not Alter the Constitutive nor the Distention-Induced Release of s-ENTDs in the LP of WT Denuded Bladders

**Figure 4:**
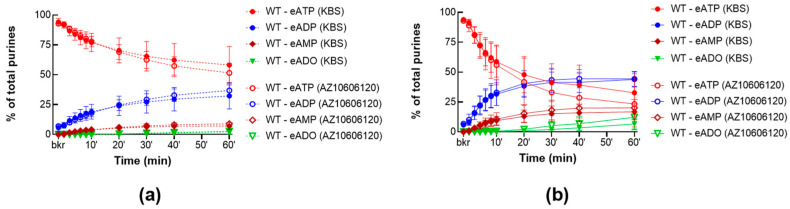
Effect of AZ10606120 on the eATP degradation by s-ENTDs released in cELS of denuded bladder preparations. Summarized data showing time courses of the degradation of eATP in cELS in the presence of the vehicle (KBS, *n* = 8–9) or AZ10606120 (25 μM, *n* = 6) in nondistended (**a**) and distended (**b**) WT bladders; *n*, number of bladder preparations. eATP, eADP, eAMP, and eADO are shown as percentages of total purines (eATP + eADP + eAMP + eADO) present in reaction solutions at each time point for the duration of 1 h.

To study the role of P2X7R in the release of s-ENTDs, we evaluated the degradation of eATP in cELS of nondistended and distended WT bladder preparations treated with AZ10606120 (25 µM), a specific antagonist of P2X7R [54]. As shown in Figure 4, AZ10606120 did not change the degradation of eATP in cELS of nondistended (Figure 4a) and distended (Figure 4b) preparations, suggesting that P2X7R might not have a physiological role in the spontaneous or distension-induced release of s-ENTDs in the LP.

#### 2.2.2. P2X7R Activation with BzATP Increases the Release of s-ENTDs in the LP of WT Bladders and Eliminates the Mechanosensitive Pattern of s-ENTDs Release

**Figure 5:**
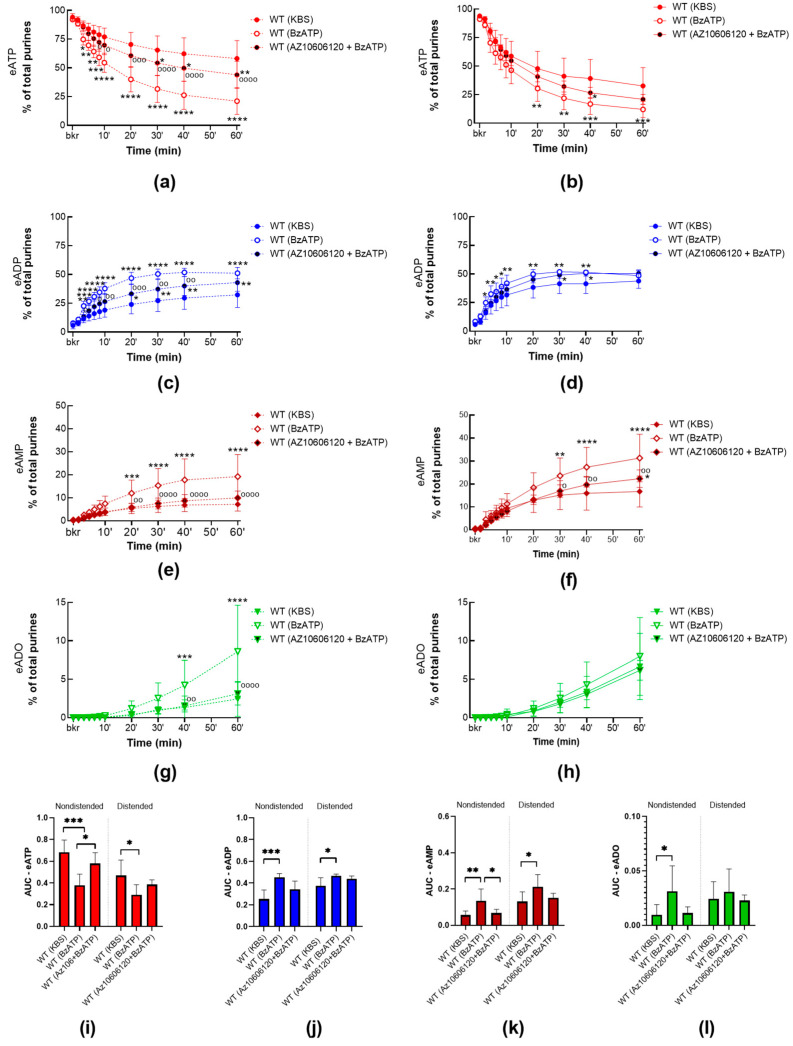
eATP degradation by s-ENTDsin cELS of WT bladders in the presence of BzATP alone or with AZ10606120. Summarized data showing time courses of eATP decrease (**a**,**b**), eADP increase (**c**,**d**), eAMP increase (**e**,**f**), and eADO increase (**g**,**h**) in cELS of nondistended (**left**) and distended (**right**) WT bladder preparations in the presence of the vehicle (KBS, *n* = 8–9), BzATP (30 µM, *n* = 5) or AZ10606120 (10–25 µM) plus BzATP (*n* = 6);* n*, number of bladder preparations. eATP, eADP, eAMP, and eADO are shown as percentages of total purines (eATP + eADP + eAMP + eADO) present in reaction solutions at each time point for 1 h. Asterisks denote significant differences from vehicle control. * *p* < 0.05, ** *p* < 0.01, *** *p* < 0.001, **** *p* < 0.0001. Open circles denote significant differences in eATP degradation in BzATP alone vs. AZ10606120 + BzATP. ^o^ *p* < 0.05, ^oo^ *p* < 0.01, ^ooo^ *p* < 0.001, ^oooo^ *p* < 0.0001. Two-way ANOVA with Tukey’s multiple comparisons test. Mean area under the curve (AUC) values for time courses of eATP (**i**), ADP (**j**), AMP (**k**), ADO (**l**). Asterisks denote significant differences between groups. * *p* < 0.05, ** *p* < 0.01, *** *p* < 0.001. One-way ANOVA with Tukey’s multiple comparisons test.

To assess whether P2X7R activation modulates the release of s-ENTDs, we compared the degradation of eATP in cELS of detrusor-free preparations isolated from WT mice in the presence or absence of BzATP (30 µM), a potent agonist of P2X7R. As shown in Figure 5, activation of P2X7R with BzATP increased the degradation of eATP and formation of eADP, AMP, and ADO in cELS of both nondistended WT bladders and distended WT bladders. Note that in cELS of nondistended preparations incubated with BzATP, almost half of the eATP was hydrolyzed in the first 10 min of the reaction and only about 20% was left at the end of the reaction (vs. 58% in controls). The AUC of eATP decreased roughly by half in nondistended preparations and by 40% in distended preparations incubated with BzATP when compared with controls. This suggests that BzATP greatly increased the release of s-ENTDs at rest and, to a lesser degree, the distension-induced release of s-ENTDs, which eliminated the mechanosensitive pattern observed in controls.

To verify whether BzATP-induced release of s-ENTDs was primarily due to activation of P2X7R, BzATP was applied in bladder preparations pretreated with the P2X7R antagonist AZ10606120. The effect of BzATP in nondistended and distended WT bladder preparations was attenuated by the inhibition of P2X7R by AZ10606120 (Figure 5). Thus, in cELS of nondistended preparations incubated with both AZ10606120 and BzATP, the eATP decrease was diminished when compared with those incubated with BzATP alone, resulting in twice as much eATP left at the end of the reaction (Figure 5a). In cELS of distended preparations incubated with both AZ10606120 and BzATP, the eATP decrease showed a trend to be lower than observed for distended preparations incubated with BzATP alone, but this difference did not reach statistical significance (Figure 5b). Nevertheless, in the last 30 min of reaction, the increase in eAMP was significantly lower (*p* < 0.05) when in the presence of both AZ10606120 and BzATP, rather than in the presence of BzATP alone.

These data further support that BzATP-induced release of s-ENTDs into the LP occurs via P2X7R activation.

#### 2.2.3. High Concentrations of ATP Facilitate the Release of s-ENTDs in the LP of WT Bladders

**Figure 6:**
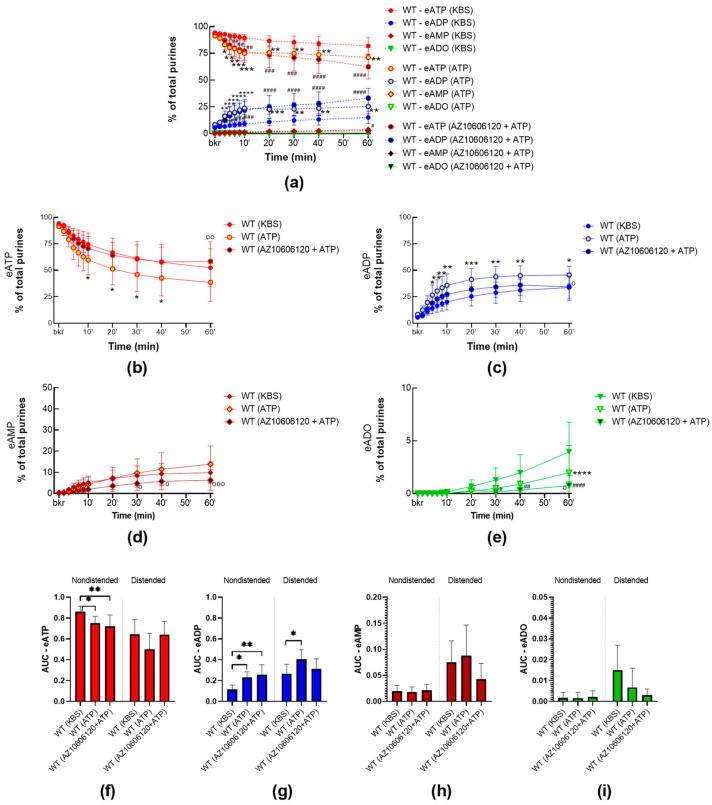
Hydrolysis of eATP by nucleotidases-ENTDs released in cELS of bladder preparations from WT mice at rest and during filling in the presence of ATP alone or with AZ10606120. Summarized data showing time courses of the degradation of eATP in cELS of nondistended (**a**) and distended (**b**–**e**) WT bladder preparations in the presence of the vehicle (KBS, *n* = 9), ATP (3 mM, *n* = 5) or AZ10606120 (25 µM) plus ATP (*n* = 6); *n*, number of bladder preparations. eATP, eADP, eAMP, and eADO are shown as percentages of total purines (eATP + eADP + eAMP + eADO) present in reaction solutions at each time point for 1 h. Asterisks denote significant differences in ATP 3 mM from the vehicle. Hash symbols denote significant differences in ATP 3 mM with AZ10606120 from the vehicle. * *p* < 0.05, ** *p* < 0.01, *** *p* < 0.001, **** *p* < 0.0001. Open circles denote significant differences in ATP alone vs. AZ10606120 + ATP. ^o^ *p* < 0.05, ^oo^ *p* < 0.01, ^ooo^ *p* < 0.001. Hash signs denoste significant differences in AZ10606120 + ATP vs. vehicle (KBS). # *p* < 0.5, ## *p* < 0.01, ### *p* < 0.001, #### *p* < 0.0001. Two-way ANOVA with Tukey’s multiple comparisons test. Mean area under the curve (AUC) values for time courses of eATP (**f**), ADP (**g**), AMP (**h**), and ADO (**i**). Asterisks denote significant differences between groups. * *p* < 0.05, ** *p* < 0.01. One-way ANOVA with Tukey’s multiple comparisons test.

To assess whether high concentrations of ATP, required to activate P2X7R, modulate the s-ENTDs release, we compared the degradation of eATP in cELS of detrusor-free WT preparations in the presence or absence of ATP (3 mM) (Figure 6). The decrease in eATP and increase in eADP in cELS of nondistended and distended preparations incubated with ATP were greater than in controls, whereas the increase in eAMP remained unchanged by ATP. The increase in eADO only reached a statistical difference from controls at 60 min in cELS of distended bladders. This suggests that release of s-ENTDs was increased in the presence of ATP in nondistended and distended bladders.

To investigate whether the ATP-induced release of s-ENTDs was due to the activation of P2X7, ATP was applied in the presence of AZ10606120. AZ10606120 did not attenuate ATP effect in nondistended bladders, but it diminished the effect of ATP in distended bladders (Figure 6b–e). As such, in cELS from distended preparations incubated with both AZ10606120 and ATP, the eATP decrease and eADP increase were not different from vehicle controls. However, at 60 min of reaction, the degradation of eATP in the presence of AZ10606120 was significantly lower (*p* = 0.0093) than in cELS of distended preparations treated with ATP. These data suggest that high concentrations of ATP facilitate the release of s-ENTDs into the LP and that this effect is in part mediated through the activation of P2X7R.

### 2.3. Interdependence of PANX1 and P2X7R in Mediating s-ENTDs Release in LP

#### 2.3.1. BzATP Has no Effect on the Release of s-ENTDs in the LP of *Panx1^−/−^* Detrusor-Free Bladders

**Figure 7:**
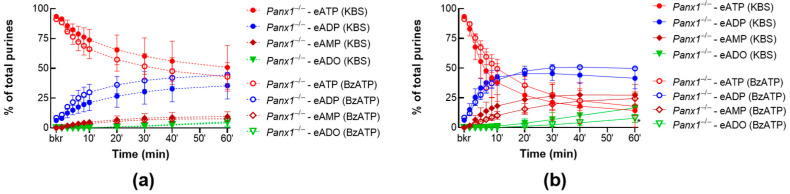
eATP hydrolysis by s-ENTDs released in cELS of *Panx1^−/−^* bladder preparations in the presence of BzATP. Summarized data showing time-courses of the degradation of eATP in cELS of nondistended (**a**) and distended (**b**) *Panx1^−/−^* bladder preparations in the presence of vehicle (KBS, *n* = 7) or BzATP (30 µM, *n* = 4). Asterisks denote significant differences from vehicle controls. * *p* < 0.05; two-way ANOVA with Sidak’s multiple comparisons test.

To examine whether the effect of P2X7R activation is reliant on interaction with PANX1, we compared the degradation of eATP in cELS of detrusor-free bladder preparations isolated from *Panx1***^−/−^** mice in the presence or absence of BzATP (30 µM). BzATP did not affect the release of s-ENTDs in nondistended and distended *Panx1***^−/−^** bladders (Figure 7a,b). As such, the decrease in eATP and the increase in its e-metabolites in cELS from *Panx1***^−/−^** preparations incubated with BzATP were not significantly different (*p* > 0.05) from *Panx1***^−/−^** controls. Note that the eATP decrease was lower in cELS of nondistended *Panx1***^−/−^** preparations incubated with BzATP than in nondistended WT preparations incubated with BzATP (eATP: ~43% and 21% of the total of purines at 60 min, respectively; *p* = 0.0017). These data suggest that P2X7R-PANX1 interaction is essential for the BzATP-evoked release of s-ENTDs. 

#### 2.3.2. BzATP-Induced Release of s-ENTDS Is Blocked in the Presence of ^10^Panx

**Figure 8:**
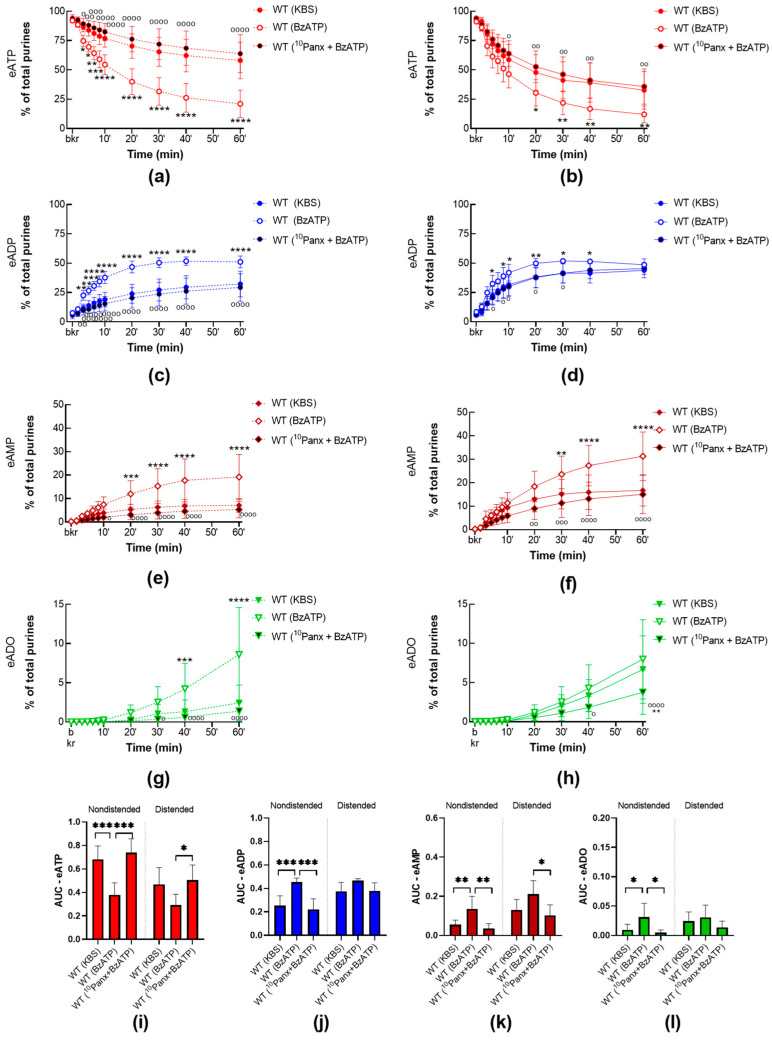
Degradation of eATP by s-ENTDsin cELS of WT bladder preparations in the presence of BzATP alone or with ^10^Panx. Summarized data showing time courses of eATP decrease (**a**,**b**), eADP increase (**c**,**d**), eAMP increase (**e**,**f**), and eADO increase (**g**,**h**) in cELS of nondistended (**left**) and distended (**right**) WT bladder preparations in the presence of the vehicle (KBS, *n* = 8–9), BzATP (30 µM, *n* = 5) or ^10^Panx (200 µM) plus BzATP (30 µM, *n* = 5); *n*, number of bladder preparations. eATP, eADP, eAMP, and eADO are shown as percentages of total purines (eATP + eADP + eAMP + eADO) present in reaction solutions at each time point for the duration of 1 h. Asterisks denote significant differences vs. vehicle control. * *p* < 0.05, ** *p* < 0.01, *** *p* < 0.001, **** *p* < 0.0001. Open circles denote significant differences between BzATP alone and ^10^Panx plus BzATP. ^o^ *p* < 0.05, ^oo^ *p* < 0.01, ^ooo^ *p* < 0.001, ^oooo^ *p* < 0.0001. Two-way ANOVA with Tukey’s multiple comparisons test. Mean area under the curve (AUC) values for time courses of eATP (**i**), ADP (**j**), AMP (**k**), ADO (**l**). Asterisks denote significant differences between groups. * *p* < 0.05, ** *p* < 0.01, *** *p* < 0.001. One-way ANOVA with Tukey’s multiple comparisons test.

Next, to investigate whether BzATP-evoked release of s-ENTDs was dependent on PANX1 channel activity, BzATP (30 µM) was applied together with ^10^Panx (200 µM), a peptide inhibitor of PANX1. Although incubation with ^10^Panx alone had no effect on the degradation of eATP by released s-ENTDS as shown in Figure 3, ^10^Panx prevented the BzATP effect in cELS of nondistended and distended WT bladder preparations (Figure 8). As such, in cELS preparations incubated with ^10^Panx and BzATP together, the eATP decrease and increase in eADP, eAMP, and ADO were not different from controls (vehicle) but were significantly different (*p* < 0.05) from preparations incubated with BzATP alone. This suggests that PANX1 channel activity is critical for the facilitated release of s-ENTDS in response to P2X7 activation.

#### 2.3.3. ATP-Induced Release of s-ENTDs during Distension Is Diminished in *Panx1^−/−^* Bladders

**Figure 9:**
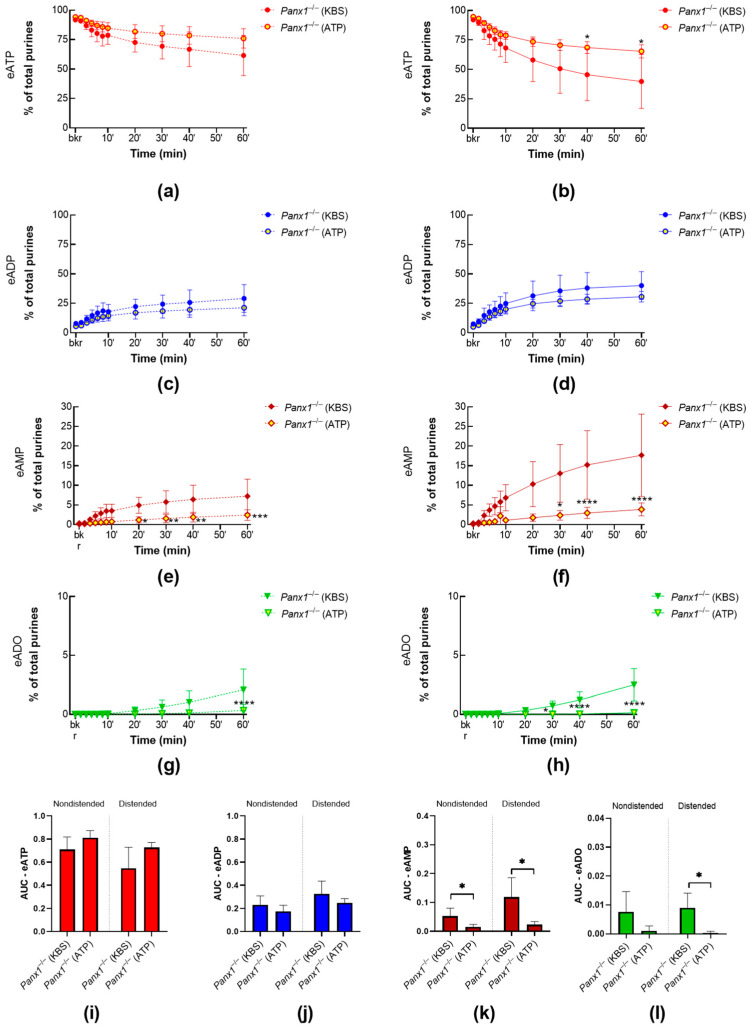
Hydrolysis of eATP by s-ENTDs released in cELS of bladder preparations from *Panx1^−/−^* mice at rest and during filling in the presence of ATP. Summarized data showing time courses of the degradation of eATP in cELS of nondistended (**a**,**c**,**e**,**g**) and distended (**b**,**d**,**f**,**h**) *Panx1^−/−^* bladder preparations in the presence of the vehicle (KBS, *n* = 5) or ATP (3 mM, *n* = 4);* n*, number of bladder preparations. eATP, eADP, eAMP, and eADO are shown as percentages of total purines (eATP + eADP + eAMP + eADO) present in reaction solutions at each time point for the duration of 1 h. Asterisks denote significant differences in ATP 3 mM from the vehicle. * *p* < 0.05, ** *p* < 0.01, *** *p* < 0.001, **** *p* < 0.0001. Two-way ANOVA with Tukey’s multiple comparisons test. Mean area under the curve (AUC) values for time courses of eATP (**i**), ADP (**j**), AMP (**k**), ADO (**l**). Asterisks denote significant differences from *Panx1*^−/−^ (vehicle). * *p* < 0.05; unpaired *t* test.

To assess whether high concentrations of ATP require PANX1 to modulate the release of s-ENTDs, we compared the degradation of eATP in cELS of detrusor-free *Panx1^−/−^* bladder preparations in the presence or absence of ATP (3 mM) (Figure 9). The decrease in eATP and the increase in e-products in cELS of distended preparations were reduced. Similarly, the degradation of eATP in nondistended preparations appeared to be reduced by exogenous ATP; however, statistical significance was reached in the formation of eAMP and eADO only.

## 3. Discussion

The urinary bladder’s main functions to store and void urine are under complex neural control and local modulation at the mucosa-detrusor interface (i.e., LP). The current notion is that in response to bladder distention during filling, the urothelium releases ATP into the LP, which then activates sensory neurons residing in LP and urothelium and triggers the micturition reflex [55]. Therefore, extracellular ATP is recognized as a key regulator of bladder excitability [4]. The mechanisms by which effective concentrations of ATP at receptor sites in sensory neurons or other cell types in LP are maintained are not fully understood. We recently demonstrated that the degradation of ATP in the LP is a mechanosensitive process that is mediated by mb-ENTPDs and s-ENTPDs [5]. s-ENTDs, in particular, provide means to regulate precisely the effective concentrations of ATP in the extracellular space in LP [5,56]. Moreover, the release of s-ENTDs in the bladder LP emerges as a highly regulated process that is operated by complex systems involving sensory neurons, mechanosensitive channels, and cell surface receptors [56,57]. In the present study, we investigated whether two key players in purinergic signaling—the PANX1 channel and the P2X7R—regulate the constitutive and/or distention-induced release of s-ENTDs to maintain proper extracellular purine concentrations in the LP. We found that PANX1 and P2X7R regulate the release of s-ENTDs in LP in opposite directions while depending on each other.

PANX1 has been detected in all layers of the bladder, with greater expression in the urothelium [8,9,58]. PANX1 activation in response to mechanical stretch or changes in pressure has been well described [9,18,26]. Furthermore, mechanosensors, such as TRPV4 [59] and PIEZO1 [60] channels, have been implicated in the transduction of mechanical stretch leading to the activation of PANX1. Therefore, it is conceivable to expect that PANX1, when activated during bladder filling, will contribute to mechanotransduction in the bladder wall. Indeed, PANX1 has been proposed to mediate the release of ATP in the bladder lumen in response to bladder distension [8,9]. However, no information is available about the possible contribution of PANX1 in the control of enzyme release. In this study, we determined that deletion of *Panx1* had no effect on the constitutive release of s-ENTDs, but it did substantially increase the distension-induced release of s-ENTDs. This suggests that distention-induced activation of PANX1 during bladder filling would limit the release of these enzymes, likely preserving ATP at effective concentrations near cell surfaces at the end of bladder filling. Therefore, distention-induced activation of PANX1 in the bladder LP likely results in the release of both ATP and s-ENTDs. This scenario suggests that obtaining proper extracellular concentrations of ATP and other purine mediators at receptor sites is under rather sophisticated regulation. The observations suggesting that activation of PANX1 could restrain the distention-induced release of s-ENTDs in LP are particularly intriguing since they emulate recent findings indicating that activation of sensory neurons and PIEZO channels in response to distention confine the excessive release of s-ENTDs [56], likely to preserve ATP at effective concentrations in the vicinity of its receptors. In contrast to the effect of *Panx1* deletion that was confirmed with end-point PCR analysis, ^10^Panx, a 10-residue peptide inhibitor of PANX1 [10], had no effect on s-ENTDS release into the LP. This lack of effect of ^10^Panx may be attributed to incomplete inhibition of the PANX1 channel in a multicellular system such as the bladder mucosa.

PANX1 appears to interact functionally with the P2X7R [8], which is expressed throughout the bladder wall, including the bladder mucosa [8,61,62]. Hence, we next sought to investigate the effect of P2X7R activation on the spontaneous and distention-induced release of s-ENTDs in the LP. AZ10606120, a potent and selective antagonist of P2X7R [54], when applied alone, had no effect on the release of s-ENTDs in the LP of empty or filled preparations. It is possible that physiological activation of P2X7R in response to mechanical stimulation, as proposed by Negoro and colleagues [8], does not participate in the regulation of s-ENTDs release or that AZ10606120 is not as selective and potent inhibitor of the P2X7R as originally suggested. We provide evidence that the latter possibility is less likely to occur since AZ10606120 considerably reduced the effects of a P2X7R agonist (discussed below). In contrast to the lack of effect of the P2X7R antagonist, activation of P2X7R with BzATP, an agonist of P2X7R with 10–30-fold greater potency than ATP [38], greatly increased the release of s-ENTDs in nondistended WT bladders and, to a lesser extent, the release in distended bladders, eliminating the mechanosensitive pattern of s-ENTD release observed in controls. AZ10606120 attenuated the BzATP-induced release of s-ENTDs and restored the mechanosensitive pattern of s-ENTD release. These results confirmed that (1) AZ10606120 is indeed an inhibitor of the P2X7R in our system, and (2) the BzATP effect we observed most likely resulted from the activation of P2X7R. 

P2X7R may be naturally activated by high concentrations of ATP [38]. These higher concentrations of ATP may be available during inflammation, in tumor microenvironments, or when nucleotidases are downregulated [63]. In particular, the increased urothelial release of ATP was described in patients with interstitial cystitis/bladder pain syndrome [64,65], overactive bladder [66], and voiding dysfunction secondary to benign prostatic hyperplasia [67], as well as in animal models of bladder dysfunction [68,69,70,71,72]. In the present study, exogenously applied ATP (3 mM) facilitated the release of s-ENTDs into the LP of both nondistended and distended bladders. These effects of ATP were similar to the effects of the P2X7R agonist BzATP. Similarly, AZ10606120 attenuated the effect of ATP in distended preparations, suggesting that the effect of ATP on the release of s-ENTDs was at least in part mediated via P2X7R. Therefore, we propose that activation of P2X7R, likely occurring as part of the inflammatory process of cystitis, would facilitate s-ENTD-mediated ATP hydrolysis and prevent disproportionate bladder excitability caused by abnormally high extracellular ATP.

P2X7R activation can induce cytoskeletal rearrangements, such as blebbing and/or shedding of microvesicles in macrophages, monocytes, and HEK293 cells transfected with the rat P2X7R receptor [73,74,75]. Although we have not investigated whether the shedding of microvesicles in response to P2X7R activation occurs in our bladder preparations, this could be a mechanism facilitating the release of enzymes. Noteworthy, the release of ENTDS through microvesicles or exosomes has been previously described [76,77,78]. Furthermore, our finding that P2X7R modulates the release of nucleotidases in the LP of the bladder is consistent with the notion that P2X7R activation can induce the release of cellular proteins. Accordingly, this receptor has been found to participate in the release of IL-B1 [79] and other interleukins, cytokines, TNF-alfa, and PGE_2_ [80,81]. It has also been suggested that P2X7R regulates neurotransmitter release [82] and synaptic vesicle release [83]. 

Interestingly, we found that BzATP had no effect in *Panx1*^−/−^ bladder preparations, which indicates that interaction with PANX1 is required for P2X7R activity with regard to s-ENTDs release. Similarly, the facilitating effect of ATP on s-ENTDs release (as seen in WT preparations) was lost in *Panx1^−/−^* preparations. What is more, the effect of exogenous ATP was even reversed so that exogenous ATP diminished the release of s-ENTDs. This observation was unexpected and might suggest that P2 receptors, in addition to the P2X7R, also participate in the regulation of enzyme release. Further studies should test this possibility. In the present study, the effect of BzATP was also lost in the presence of ^10^Panx. Together, these observations suggest that activation of P2X7R leading to s-ENTD release is dependent on PANX1 channel opening. PANX1-P2X7R cross-talk is well documented in many systems. Thus, the physical interaction of PANX1 with P2X7R has been shown through co-immunoprecipitation [10,11,12] and proximity ligation assay [13,14,15]. The functional coupling has also been shown pharmacologically or by using genetically modified mice deficient in PANX1 or P2X7R [8,14,16]. The present study provides novel insight into PANX1-P2X7 interplay and their role in modulating s-ENTDs release and, hence, ATP hydrolysis in the extracellular space. Further studies are warranted to unravel the exact mechanisms of PANX1 and P2X7R interdependence with regard to s-ENTDs release.

In summary, here we report that PANX1 activated during bladder filling likely hinders the release of s-ENTDs to ensure effective concentrations of ATP at P2 purinergic receptors. Conversely, P2X7R activation, to be expected in inflammatory conditions of the urinary bladder, augments the release of s-ENTDs, leading to greater ATP degradation, and therefore counteracting the increased bladder excitability induced by high extracellular ATP. Although PANX1 and P2X7R modulate the constitutive and distension-evoked release of s-ENTDs in opposite directions, P2X7R activity was found to be dependent on interaction with PANX1, seemingly requiring the opening of this channel. This complex interplay between PANX1 and P2X7R in the LP of the bladder might be important to ensure optimal bladder excitability. In conclusion, the present study provides strong support for the idea that a highly regulated homeostatic mechanism guarantees optimal concentrations of ATP and other purine mediators at receptor sites in the bladder LP [5,56]. Ultimately, the regulatory mechanisms involved in the release of s-ENTDs in the bladder, including pathways associated with PANX1 and P2X7R, could be targeted to improve bladder function.

## 4. Materials and Methods

### 4.1. Animals

Male and female C57BL/6J mice (wild-type controls, WT, JAX stock #000664) and *Panx1^−/−^* mice (generated in house by crossing B6;129-*Casp4^del^Panx1^tm1Vshe^,* JAX stock #026021 with *B6.C-Tg(CMV-cre)1Cgn/J*, JAX stock #006054, Jackson Laboratory, Bar Harbor, MN, USA), 12 to 24 weeks old, were sedated with isoflurane (AErrane; Baxter, Deerfield, IL, USA) and euthanized by cervical dislocation and exsanguination. Urinary bladders were excised and placed in oxygenated ice-cold Krebs-bicarbonate solution (KBS; composition in mM: 118.5 NaCl, 4.2 KCl, 1.2 MgCl_2_, 23.8 NaHCO_3_, 1.2 KH_2_PO_4_, 11.0 dextrose, and 1.8 CaCl_2_; pH 7.4) for further dissection. 

### 4.2. Ethical Approval

Animals were maintained and experiments were conducted in conformity with the National Institutes of Health Guide for the Care and Use of Laboratory Animals and the Institutional Animal Use and Care Committee at the University of Nevada, Reno.

### 4.3. RNA Isolation, Reverse Transcription, and RT-PCR

Total RNA was isolated from the whole tissue of the brain, bladder urothelium, and detrusor from WT and *Panx1*^−/−^ mice using Direct-zol™ RNA MiniPrep kit (Zymo, Irvine, Ca, USA) and eluted in 25 µL of nuclease-free water. Concentration and purity of RNA were measured using a NanoDropOne Spectrophotometer (Thermo Fisher Scientific, Waltham, MA, USA), and comparative amounts of RNA were used for first-strand cDNA synthesized using qScript™ cDNA SuperMix (Quantabio, Beverly, MA, USA), according to the manufacturer’s instructions. PCR was performed with specific primers using GoTaq^®^ Green Master Mix (Promega, Madison, WI, USA). *Panx1* primer sequences were designed in the knockout region of exons 3–4. Sequences given in parentheses were used: *Panx1* (F-GTGACTGAGAATGTGGGGCA, R-CCAGCCGGCAGCTAATGTAT), *Panx2* (F-AAAAGCATACCCGCCACTTC, R-GGAGTGGAGCATCTTTGGTG), and *Panx3* (F-CCTCACAAGGCTCTTCCCTA, R-GCGGATGGAACGGTTGTAAG). A total of 5 µL of PCR products were examined by electrophoresis at 100 V for 20 min on 2% agarose gel in 1× TAE Buffer and visualized by ethidium bromide. The marker used was DNA ladder 100 bp (Thermo Fisher, Waltham, MA, USA).

### 4.4. Detrusor-Free Bladder Preparation

After cleaning the fat and connective tissue of ex vivo bladders, the detrusor muscle was carefully removed as previously described [2,5,84]. Bladder preparations were catheterized through the urethra with a PE-20 tubing.

### 4.5. Soluble/Releasable Nucleotidase Activity in the Lamina Propria of Detrusor-Free Bladder Preparations of WT and Panx1^−/−^ Mice

The general protocol to assess soluble/releasable nucleotidase activity in the lamina propria of detrusor-free bladder preparations was performed as previously described [5]. Detrusor-free bladders from WT and *Panx1*^−/−^ mice were placed in a 3 mL chamber filled with KBS at 37 °C and bubbled with 95%O_2_/5%CO_2_. The bladder catheters were connected to an infusion pump (Genie Touch, Kent Scientific, Torrington, CT, USA) for bladder filling. Bladder preparations were equilibrated for 20 min. The bath solution was then replaced with fresh KBS, and each bladder was left empty/nondistended for the equivalent time of filling. Then, 2.9 mL of the bathing solution (designated as an extraluminal solution, ELS) was collected to a 4 mL Amicon Ultra Centrifugal Filter Unit with a 10 kDa molecular weight cut-off (MWCO) pore size (Millipore Sigma, Burlington, MA, USA). The bath was replaced with fresh KBS, and the bladder preparations were filled with KBS at 15 µL·min^−1^ to pre-voiding volume (distended condition), as previously described [2,5]. Next, 2.9 mL ELS was collected to a 4 mL Amicon Ultra Centrifugal Filter Unit with a 10 kDa MWCO. Samples were concentrated by centrifugation at 4000× *g* for 25 min at 4 °C using a swing bucket rotor (ThermoFisher Scientific SorvallST 40R, Langenselbold, Germany). The concentrated EL solutions (cELS) were brought up to 200 µL with oxygenated KBS. 1,*N*^6^-etheno-derivative of ATP (eATP; 2 µM working concentration) was used as a substrate, and enzymatic reactions were performed at 37 °C. The working concentration was within the range of the estimated concentration for total purines available in the SubU/LP surface at the end of denuded bladder filling [2]. Following the addition of eATP (0 min), 20 µL samples were collected from the reaction solution at 10 s, 2 min, 4 min, 6 min, 8 min, 10 min, 20 min, 30 min, 40 min, and 60 min, and then diluted 10-fold in ice-cold citric phosphate buffer (pH 4.0) to stop the enzymatic reactions. Collected samples were compared with 2 µM eATP in KBS that had not been in contact with any enzymes (designated as “beaker” sample). Substrate decrease and product increase were detected by HPLC-FLD methodology as described in Section 4.8.

### 4.6. Effect of Pharmacological Activation or Inhibition of P2X7R and/or PANX1 on the Release of Soluble Nucleotidases in the LP

To assess the effects of pharmacological inhibition of PANX1 or P2X7R, experiments were conducted as described in Section 4.5, where bladder preparations from WT mice were incubated with a peptide inhibitor of PANX1, ^10^Panx (200 µM), or the P2X7R inhibitor AZ10606120 (25 µM) throughout the experiment.

To assess the effect of P2X7R activation on the release of s-ENTDs in the LP, experiments were conducted as described in Section 4.5, but bladder preparations from WT or *Panx1*^−/−^ mice were incubated with the P2X7R agonist BzATP (30 µM) or ATP (3 mM) during the nondistended and distended conditions. Additionally, to ensure the return of the receptor to its basal state between nondistended and distended conditions, we performed three washes of 5 min each with oxygenated KBS and left bladder preparations for an additional 10 min in an oxygenated KBS bath. It should be noted that in contrast to other P2X receptors, P2X7 showed a complete lack of desensitization [85]. ELS of bladder preparations incubated with BzATP was concentrated, then time courses of enzymatic reactions in the presence of BzATP were performed, as described in Section 4.5. However, to avoid the appearance of chromatography peaks representing the authentic ATP that could mask the signals of eATP and its metabolites, the enzymatic reactions needed to be performed in solutions that contained released nucleotidases but no ATP. Thus, 2.9 mL ELS of bladder preparations incubated with ATP were placed in centrifugal filter units and centrifuged at 4000× *g* for 25 min at 4 °C. Then, 2.9 mL of KBS was added to the centrifugal filter units, and the samples were centrifuged again at 4000× *g* for 15 min (4 °C). Last, an additional 2.9 mL of KBS was added to the centrifugal units and concentrated at 4000× *g* for 25 min (4 °C). Time courses of enzymatic reactions in the presence of KBS were performed as described in Section 4.5. The hydrolysis of eATP in cELS, containing enzymes released in the presence of ATP, was compared with eATP hydrolysis in regular KBS processed in the same fashion.

To confirm the specificity of P2X7R activation by BzATP or ATP, similar experiments were performed where bladder preparations from WT mice were incubated with the P2X7R inhibitor AZ10606120 (10 µM or 25 µM) throughout the experiment whereas the P2X7R agonists BzATP (30 µM) or ATP (3 mM) were added to the chambers during nondistended and distended conditions.

To investigate whether the BzATP effect was dependent on PANX1 channel activity, experiments were performed where bladder preparations from WT mice were incubated with the PANX1 inhibitor ^10^Panx (200 µM) throughout the experiment whereas BzATP (30 µM) was added to the chambers during nondistended and distended conditions.

### 4.7. Preparation of 1,N^6^-Etheno-Nucleotides

1,*N*^6^-etheno-ATP (eATP) was prepared as described previously [5]. Briefly, 0.2 mM ATP was acidified with citrate phosphate buffer to pH 4.0. eATP was formed by adding 2-Chloroacetaldehyde (1 M) to acidified ATP and heating the samples to 80 °C for 40 min [86,87]. The reaction solution contained eATP at a concentration of 2 µM.

### 4.8. HPLC Analysis of 1,N^6^-Etheno-Nucleotides

A reverse-phased gradient Agilent 1200 liquid chromatography system coupled with a fluorescence detector (FLD) (Agilent Technologies, Wilmington, DE, USA) was used to detect 1,*N*^6^-etheno-purines as described previously [2,5,88]. 1,*N*^6^-etheno-derivatized purines were detected by fluorescence at an excitation wavelength of 230 nm and emission wavelength of 420 nm [87]. ChemStation (v. B04-03) software (Agilent Technologies) was used to analyze areas under the peaks. Amounts of eATP, eADP, eAMP, and eADO were determined from standard curves with 1,*N*^6^-etheno-derivatized purine standards (0.05–5 pmol).

### 4.9. Drugs and Reagents

Adenosine, ATP, ADP, AMP, and chloroacetaldehyde dimethyl acetal were purchased from Sigma-Aldrich, St. Louis, MO, USA. AZ10606120 dihydrochloride (*N*-[2-[[2-[(2-Hydroxyethyl)amino]ethyl]amino]-5-quinolinyl]-2-tricyclo[3.3.1.13,7]dec-1-ylacetamide dihydrochloride) and BzATP triethylammonium salt (2′(3′)-*O*-(4-Benzoylbenzoyl)adenosine-5′-triphosphate tri(triethylammonium) salt) were purchased from Bio-Techne Tocris, Minneapolis, MN, USA. ^10^Panx (L-tryptophyl-L-arginyl-L-glutaminyl-L-alanyl-L-alanyl-L-phenylalanyl-L-valyl-L-α-aspartyl-L-seryl-L-tyrosine, trifluoroacetate salt) was purchased from Cayman Chemical Company, Ann Arbor, MI, USA.

### 4.10. Statistical Analysis

Data values are presented as means ± SD. Means are compared by two-way ANOVA for comparison of more than two groups followed by Tukey’s or Sidak’s multiple comparisons tests per GraphPad Prism, v.8.4.2. (GraphPad Software, Inc., San Diego, CA, USA). In addition, the area under the curve (AUC) for each experiment was calculated using GraphPad Prism, which computes the area using the trapezoid rule. AUC values range between 0 and 1, as both fractions of total of purines and time in hours range from 0 to 1. AUC values from two groups or more are compared by unpaired *t*-test or one-way ANOVA, respectively. A probability value less than 0.05 was considered statistically significant. Please note that this is an exploratory study [89], hence the calculated *p*-values are descriptive and should not be interpreted as hypothesis testing.

Parts of this work have been previously presented in abstract form at the Society of Urodynamics, Female Pelvic Medicine & Urogenital Reconstruction, SUFU 2023 Winter Meeting, Nashville, 7–11 March 2023

## Data Availability

The raw data supporting the conclusion of this article will be made available by the authors upon reasonable request. The data are not publicly available due to privacy.
